# Crystal Structures of RNase H2 in Complex with Nucleic Acid Reveal the Mechanism of RNA-DNA Junction Recognition and Cleavage

**DOI:** 10.1016/j.molcel.2010.11.001

**Published:** 2010-11-24

**Authors:** Monika P. Rychlik, Hyongi Chon, Susana M. Cerritelli, Paulina Klimek, Robert J. Crouch, Marcin Nowotny

**Affiliations:** 1 Laboratory of Protein Structure, International Institute of Molecular and Cell Biology, 4 Trojdena Street, 02-109 Warsaw, Poland; 2 Program in Genomics of Differentiation, Eunice Kennedy Shriver National Institute of Child Health and Human Development, National Institutes of Health, Bethesda, MD 20892, USA

## Abstract

Two classes of RNase H hydrolyze RNA of RNA/DNA hybrids. In contrast to RNase H1 that requires four ribonucleotides for cleavage, RNase H2 can nick duplex DNAs containing a single ribonucleotide, suggesting different in vivo substrates. We report here the crystal structures of a type 2 RNase H in complex with substrates containing a (5′)RNA-DNA(3′) junction. They revealed a unique mechanism of recognition and substrate-assisted cleavage. A conserved tyrosine residue distorts the nucleic acid at the junction, allowing the substrate to function in catalysis by participating in coordination of the active site metal ion. The biochemical and structural properties of RNase H2 explain the preference of the enzyme for junction substrates and establish the structural and mechanistic differences with RNase H1. Junction recognition is important for the removal of RNA embedded in DNA and may play an important role in DNA replication and repair.

## Highlights

► Structures of RNase H2 in complex with nucleic acid substrate reported ► The mechanism of specific recognition of (5′)RNA-DNA(3′) junction revealed ► Specific deformation of the junction leads to substrate-assisted catalysis

## Introduction

RNA/DNA hybrids are formed during DNA replication and transcription, either as part of normal processes, such as during Okazaki fragment initiation, or accidentally when nascent RNAs fail to engage with the posttranscriptional machinery and instead hybridize to DNA. Single ribonucleotides in dsDNA can result from misincorporation by DNA polymerase or incomplete removal of Okazaki fragment primers (Qiu et al., 1999a). There are three types of RNA/DNA: (1) simple RNA/DNA hybrids (one strand RNA, the other DNA), (2) RNA-DNA/DNA (as found in Okazaki primers), and (3) DNA-RNA_1-few_-DNA/DNA (one or a few ribonucleotides embedded in dsDNA). Ribonucleases H are the only known enzymes that degrade the RNA strand of RNA/DNA hybrids in a sequence-nonspecific manner, and therefore are essential for DNA integrity. RNases H are classified as types 1 and 2 (RNases H1 and RNases H2) based on sequence conservation and substrate preference (Tadokoro and Kanaya, 2009). Type 1 enzymes hydrolyze all types of RNA/DNA hybrids but require at least four ribonucleotides embedded in a dsDNA sequence to cleave (Ohtani et al., 1999), while most type 2 enzymes can hydrolyze all types of hybrids including DNA-RNA_1_-DNA/DNA (Eder and Walder, 1991; Eder et al., 1993; Jeong et al., 2004; Ohtani et al., 1999, 2008). The ability to cleave single ribonucleotides suggests RNases H2 involvement in DNA replication and repair (Arudchandran et al., 2000; Qiu et al., 1999b; Rydberg and Game, 2002). RNase H2 together with Fen1 has been shown to remove single ribonucleotides, which are misincorporated by DNA polymerases (Qiu et al., 1999b; Rydberg and Game, 2002). Recent data show that more than 10,000 such misincorporations may occur in yeast during one replication cycle (Nick McElhinny et al., 2010). In some cases, RNases H2 can also participate in the processing of Okazaki fragments, but it mainly involves Fen1 and/or Dna2 (Kao and Bambara, 2003; Qiu et al., 1999b; Rydberg and Game, 2002).

RNases H are present in all kingdoms of life, and most organisms contain both types, with the exception of some archaea that have only type 2 enzyme (Tadokoro and Kanaya, 2009). Type 1 RNase H domains are also essential parts of retroviral reverse transcriptase proteins (Champoux and Schultz, 2009). In many single-celled species, including bacteria and yeast, both RNase H1 and RNase H2 can be deleted; however, in mammals, both type 1 and type 2 RNase H have essential roles. Deletion of RNase H1 in mouse impairs mitochondrial DNA replication causing embryonic lethality (Cerritelli et al., 2003). Mutations in human RNase H2 induce Aicardi-Goutières syndrome (AGS), a genetic disorder with symptoms similar to in utero viral infection, which severely affects the nervous system by activating the innate immune system (Crow et al., 2006).

Type 2 RNase H is the main RNase H activity in human cells (Eder and Walder, 1991; Frank et al., 1998; Stein and Hausen, 1969). As first identified in *S. cerevisiae*, eukaryotic type 2 RNases H are composed of three subunits: catalytic subunit (RNase H2A) and auxiliary subunits (RNase H2B and RNase H2C) (Cerritelli and Crouch, 2009). RNase H2B has a PCNA binding site and interacts with PCNA in vitro, suggesting that the accessory subunits may serve as a platform for interaction with replication/repair complexes (Chon et al., 2009). Mutations in any of the three subunits of the human enzyme can result in AGS (Crow et al., 2006), indicating that RNase H2 defects lead to the accumulation of RNA/DNA hybrids that in turn activate innate immune response (Crow et al., 2006; Stetson et al., 2008).

RNase H belongs to the retroviral integrase superfamily (RISF). RISF comprises many important and interesting nucleic acid-processing enzymes including transposases, integrases, Argonaute, Holliday junction resolvases, and other nucleases (Nowotny, 2009). Mechanistically and structurally, RISF enzymes show remarkable similarities. They share the same fold of the catalytic core (called RNase H fold), whose main element is a five-stranded β sheet with three antiparallel and two parallel strands (Nowotny, 2009; Yang and Steitz, 1995). This β sheet is flanked by α helices of variable number and arrangement. The active site of RISF enzymes is composed primarily of aspartates and glutamates. In RNases H, the active sites form an absolutely conserved DED(D/E) motif. The first and third key aspartates are spatially conserved in both types of RNases H and among RISF members—the first one is located in the middle of the first strand of the central β sheet, the other at the end of the fourth strand. The second and fourth carboxylates are located in different parts of the catalytic cores of type 1 and type 2 enzymes. The negatively charged active sites of RNases H and other RISF proteins bind divalent metal ions that are essential for catalytic activity. Most enzymes use Mg^2+^, but other divalent metal ions such as Mn^2+^ can also support catalysis. RISF enzymes are universally inhibited by Ca^2+^.

The crystal structures of substrate complexes of catalytic domains of *B. halodurans* and human RNases H1 (Nowotny et al., 2005, 2007) revealed that substrate binding involves contacts between the enzyme and 2′-OH groups of four consecutive nucleotides of the RNA strand. The DNA strand is recognized by its ability to adopt B-form conformation. Catalysis proceeds via a two-metal ion mechanism (Steitz and Steitz, 1993; Yang et al., 2006). The crystal structures of single polypeptide archaeal and bacterial type 2 RNases H from *A. fulgidus*, *M. jannaschii*, *T. kodakaraensis* (Chapados et al., 2001; Lai et al., 2000; Muroya et al., 2001), and *T. maritima* (Protein Data Base [PDB] 2ETJ) show similarity of overall topology and fold to the catalytic core of type 1 enzymes with several insertions, such as helices between β strands 2 and 3. RNases H2 also contain an additional C-terminal helix-loop-helix domain. Recently, the structure of the trimeric eukaryotic RNase H2 from mouse was reported (Shaban et al., 2010), showing that B and C subunits adopt a tightly intertwined dimer that interacts with the catalytic subunit away from the nucleic acid interface.

We report here the crystal structures of RNase H2 in complex with substrate and describe the specific processing of a (5′)RNA-DNA(3′) junction. The structures, together with our biochemical studies, reveal the mechanism of the specific junction recognition and help explain the differences between type 1 and type 2 RNases H.

## Results and Discussion

### Purification and Biochemical Characterization of *T. maritima* RNase H2

**Figure 1:**
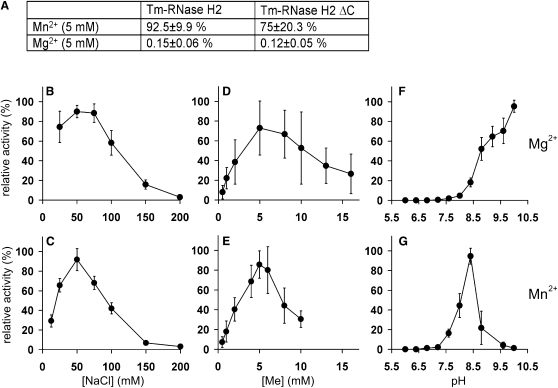
Characterization of Tm-RNase H2 Activity on Uniformly Labeled Poly(rA)/Poly(dT) Substrate (A) Mg- and Mn-dependent activities of Tm-RNase H2 (full-length and ΔC). The values represent the percent of the highest measured activity. (B–G) Dependence of Tm-RNase H2 activity on salt concentration (B and C), metal ion concentration (D and E), and pH (F and G). Upper panels show the activity in the presence of Mg^2+^ and lower panels in Mn^2+^. The activity is shown as percent of the highest measured value for each titration. Error bars represent the standard deviation of each measurement.

Expression constructs were prepared for the full-length *Thermotoga maritima* RNase H2 (Tm-RNase H2) and a variant with 15 residues removed from the C terminus (Tm-RNase H2 ΔC). These residues were not observed in the apo structure of the protein solved at the Joint Center for Structural Genomics (JCSG, PDB 2ETJ), and therefore are probably disordered. Both variants of the Tm-RNase H2 were expressed in *E. coli* and purified. The activity of both proteins was characterized using a poly(rA)/poly(dT) substrate. The full-length and C-terminally truncated versions had similar activity, and both were around 600-fold more active in the presence of Mn^2+^ compared to Mg^2+^ (Figure 1A). This is in contrast to the eukaryotic RNases H2, which are more active in the presence of Mg^2+^. The optimum NaCl concentration for activity was determined to be 50 mM (Figures 1B and 1C) and optimal metal ion concentration was around 4–7 mM, for both Mg^2+^ and Mn^2+^ (Figures 1D and 1E). The Mn-dependent activity of Tm-RNase H2 was the highest at around pH 8.5 and for Mg-dependent hydrolysis activity gradually increased from pH 7.5 to 10 without reaching a maximum (Figures 1F and 1G). The hydrolysis at high pH was protein dependent and did not occur without the enzyme added (data not shown). The dependence of Tm-RNase H2 activity on metal ion and salt concentration and the pH dependence in the presence of Mn^2+^ are similar to yeast and human enzymes (Chon et al., 2009; Rohman et al., 2008). Mg-dependent activity of Tm-RNase H2 increases with pH, which might indicate that in the presence of Mg^2+^ and RNA/DNA hybrid substrate the active site is not well organized and activation (deprotonation) of the attacking nucleophillic water is not optimal. This defect in nucleophile activation may be alleviated at higher pH.

**Figure 2:**
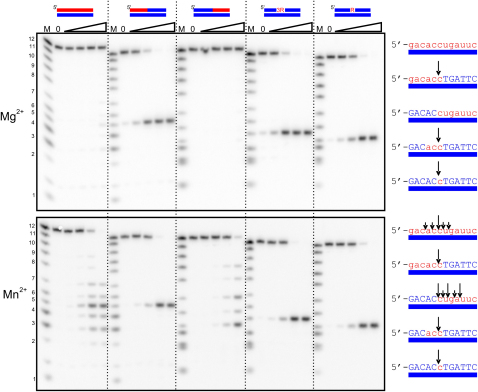
Hydrolysis of Various Substrates by Tm-RNase H2 The 5′ end ^32^P-labeled substrates (1 μM) indicated above the gel (RNA in red, DNA in blue) were hydrolyzed with increasing concentrations of Tm-RNase H2. The lanes marked with 0 contained no enzyme, and lanes marked with triangle contained increasing amounts of the protein (0.16, 1.6, 16, and 160 nM). Reaction mixtures (20 μl) were incubated at 37°C for 30 min in the presence of 1 mM MgCl_2_ (upper panel) or 1 mM MnCl_2_ (lower panel). Products of the hydrolysis were analyzed by 20% TBE-urea polyacrylamide gels. The sizes of products were measured based on molecular size markers indicated as M (products of digestion of ^32^P-labeled strands without complementary DNA by phosphodiesterase I), and the major cleavage sites are summarized at the right of each gel.

Cleavage assays were next performed for full-length Tm-RNase H2 using several 12 bp oligonucleotides all containing the same noncleaved DNA strand and different cleaved strands to form various substrates: an RNA/DNA hybrid (RNA_12_/DNA_12_), a duplex containing a (5′)RNA-DNA(3′) junction with one, three, or six ribonucleotides (DNA_5_-RNA_1_-DNA_6_/DNA_12_, DNA_3_-RNA_3_-DNA_6_/DNA_12_, and RNA_6_-DNA_6_/DNA_12_), and a substrate with a (5′)DNA-RNA(3′) junction (DNA_5_-RNA_7_/DNA_12_) (Figure 2). In the presence of Mg^2+^, there is a great preference for substrates with (5′)RNA-DNA(3′) junction, and hydrolysis occurred specifically at the 5′ side of the ribonucleotide of the junction (Figure 2). The activity was slightly lower for the substrate with a single ribonucleotide. RNA_12_/DNA_12_ hybrid and the substrate with (5′)DNA-RNA(3′) junction were very poorly cleaved. However, in the presence of Mn^2+^, all substrates were cleaved in a similar manner with no clear preference for junction substrates. RNA_12_/DNA_12_ hybrid was hydrolyzed more than 100-fold faster in the presence of Mn^2+^ compared to Mg^2+^, consistent with the results observed with poly(rA)/poly(dT). The RNA_12_/DNA_12_ hybrid and (5′)DNA-RNA(3′) substrate were cleaved at multiple sites (Figure 2), while the junction substrates were cleaved at (5′)RNA-DNA(3′). Junction substrates were hydrolyzed slightly better in the presence of Mg^2+^ than Mn^2+^.

Inside bacterial cells the total Mg^2+^ concentration is 100-fold higher than that of Mn^2+^ (Medicis et al., 1986), so for *T. maritima* RNase H2, Mg-dependent junction cleavage probably dominates over Mn-dependent RNA/DNA hydrolysis. For *T. thermophilus* RNase H2, only junction cleavage activity was reported in vitro, and it can be assumed that this is the only substrate of this protein (Ohtani et al., 2008). In contrast, eukaryotic RNases H2 in the presence of Mg^2+^ degrade junction substrates and RNA/DNA hybrids equally well (Chon et al., 2009; Rohman et al., 2008), indicating a broader range of in vivo substrates.

### Structure Determination of Tm-RNase H2-Nucleic Acid Complex

**Figure 3:**
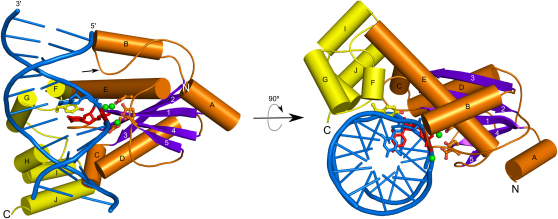
Overall Structure of Tm-RNase H2 Substrate Complex, Two Views The two domains are color coded (N-terminal domain in purple for β strands and orange for the rest of the structure and the C-terminal domain in yellow). The mobile loop (including the DSK motif) is indicated with an arrow. Nucleic acid is shown in cartoon representation with DNA in blue and the single ribonucleotide in red. Nucleic acid residues at positions +1 and +2 (the [5′]RNA-DNA[3′] junction) are shown as sticks. Active site residues are shown in orange ball and sticks and the tyrosine 163 interacting with the 2′-OH group of the ribonucleotide at the junction in yellow. Calcium ions are shown as green spheres.

For the initial crystallization trials, we employed an inactive Tm-RNase H2 D107N protein. Tm-RNase H2 ΔC D107N underwent extensive crystallization experiments in the presence of RNA/DNA hybrids of various lengths and 12 bp dsDNA harboring a single ribonucleotide in one of the strands (DNA_5_-RNA_1_-DNA_6_/DNA_12_). Protein-nucleic acid complex crystals were obtained only for the latter substrate, and they grew in the presence of 0.3 M MgCl_2_. The complex structure was solved by molecular replacement method with the apo structure solved at JCSG as a search model and subsequent manual modeling of the nucleic acid. We call this structure 1R-Mg, and it was refined to 2.0 Å resolution (Table 1). We next solved a structure from the original crystals soaked in 30 mM Mn^2+^ (structure 1R-Mn). The third structure reported here is that of wild-type ΔC protein in complex with a single ribonucleotide substrate in the presence of Ca^2+^ to inhibit cleavage, solved at 2.1 Å resolution (structure WT-1R-Ca) (Table 1). All three complexes contain one protein and one nucleic acid molecule, and their conformations are virtually identical between structures. The 5′-phosphate of the ribonucleotide of the (5′)RNA-DNA(3′) junction is located at the active site of the enzyme in agreement with the cleavage preference (Figure 3). The protein undergoes little conformational changes upon substrate binding, and its structures in the complex with nucleic acid and in the apo form are almost identical. Also in type 1 RNase H no major conformational changes are observed upon substrate binding and hydrolysis (Nowotny et al., 2005; Nowotny and Yang, 2006). Tm-RNase H2 is very similar to the other RNases H2 for which three-dimensional structures are known. For example, after superposition with *A. fulgidus* RNase H2 the root-mean-square deviation (rmsd) of the positions of 128 C-α atoms is 2.5 Å.

Tm-RNase H2 contains two domains—the N-terminal catalytic core adopts the RNase H fold (Figure 3). Its key element is the central β sheet with three longer antiparallel strands (1, 2, and 3) and two shorter ones (4 and 5) parallel to strand 1. Two helices (C and D) are located on one side of the central β sheet and three helices (A, B, and E) on the other side. In the canonical RNase H fold present in most RISF members, including RNases H1, the first three strands run without any insertions, whereas in RNases H2 there are one or two α helices inserted between strands 2 and 3 (helix B in Tm-RNase H2). The C-terminal domain of Tm-RNase H2 comprises helices F–J, and helices F and G form a helix-loop-helix motif.

### Nucleic Acid Conformation and Binding

**Figure 4:**
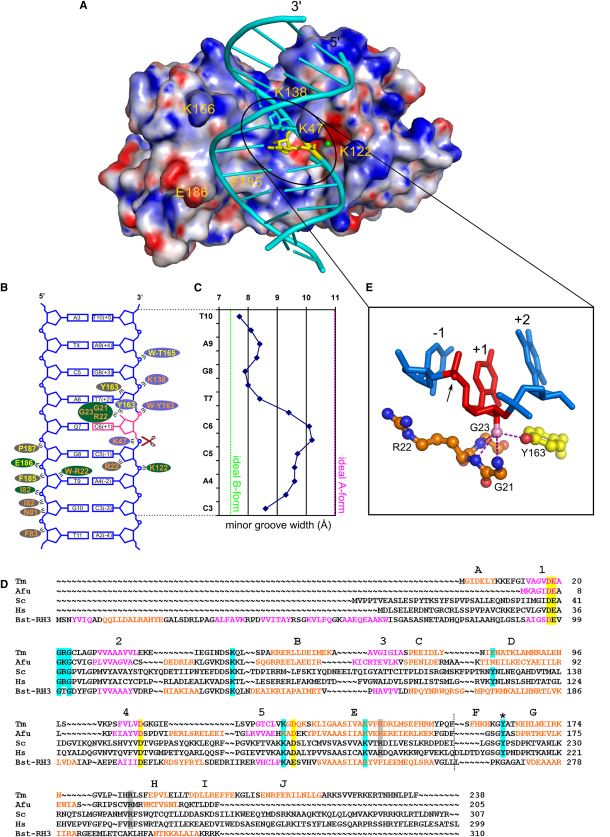
Substrate Binding (A) Surface representation of Tm-RNase H2 with electrostatic potential (±25 kT/e) coded in blue (positive) and red (negative). The nucleic acid substrate is shown in cartoon representation (single ribonucleotide in yellow) and calcium ions at the active site as green spheres (only ion C is visible). (B) Schematic representation of protein-nucleic acid contacts. The single ribonucleotide is in pink and the scissile phosphate is indicated with scissors. Residue numbers are color coded for the domains (orange for catalytic and yellow for C-terminal). The colors of ovals indicate interactions with the amino acid side chain (blue), backbone (green), or van der Waals interactions (gray). “W” before residue number denotes a water-mediated interaction. (C) Minor groove width by residue. (D) Multiple sequence alignment of selected RNase H2 sequences. RNase H3 sequence is also included for comparison. Tm, *T. maritima*; Afu, *A. fulgidus*; Sc, *S. cerevisiae*; Hs, human; Bst-RH3, *B. stearothermophilus* RNase H3. The active site residues are highlighted in yellow, selected nucleic acid binding residues in cyan, and two arginines stabilizing the GRG motif in gray. For proteins of known structure the sequence is colored according to the secondary structure (orange for helices, purple for strands). Secondary structure elements in Tm-RNase H2 are labeled on top of the alignment. The tyrosine interacting with 2′-OH is indicated with an asterisk. The boundary between the two RNase H2 domains is shown with a dashed line. (E) Close-up view of the interaction of 2′-OH (pink sphere). The nucleotides are numbered relative to the scissile phosphate, which is indicated with an arrow.

The nucleic acid substrate is bound in a groove on the surface of the protein which is formed between the catalytic and the C-terminal domains (Figure 3). This groove is overall positively charged with the exception of the carboxylate-rich active site (Figure 4A). The enzyme interacts with seven base pairs of the substrate (Figure 4B). It is bound on the minor groove side of the double helix, which is consistent with non-sequence-specific cleavage by RNase H2. The double helix of the substrate has a much shallower and wider minor groove in the region that interacts with the protein. The width of the minor groove in this region is around 10 Å, which corresponds to an A-form nucleic acid (Figure 4C). In agreement with that, at the protein interface the sugar puckers of the cleaved strand are C3′-endo, typical for A-form conformation. Outside of the interface, the nucleic acid is more B-form with narrow and deep minor groove and corresponding B-form sugar puckers. The fact that the substrate is predominantly A-form at the complex interface explains why some RNases H2, for example *T. thermophilus* enzyme, are able to cleave substrates with (5′)RNA-DNA(3′) junction surrounded by dsDNA regions which can adopt both A-form and B-form conformations, as well as those with dsRNA regions whose conformation is restricted to A-form nucleic acid (Ohtani et al., 2008).

For the noncleaved strand, a larger portion of the protein-substrate interactions are with the C-terminal domain and are either van der Waals contacts or are mediated by the backbone of the protein (Figure 4B). This suggests that the molecular shape of the protein surface is mostly responsible for the binding of the noncleaved strand. The cleaved strand containing the single ribonucleotide interacts mostly with the catalytic domain (Figure 4B). Several charged side chains participate in the binding. For example K47, K122, and K138 form contacts with the phosphate groups of the nucleic acid backbone. The three residues are strongly conserved in RNases H2 from bacteria to human (Figure 4D). Mutation of K138 equivalent in *A. fulgidus* RNase H2 led to a 20-fold decrease in activity (Chapados et al., 2001). The conserved R22 residue inserts itself into the minor groove of the substrate, and its long side chain is positioned along the minor groove.

**Figure 5:**
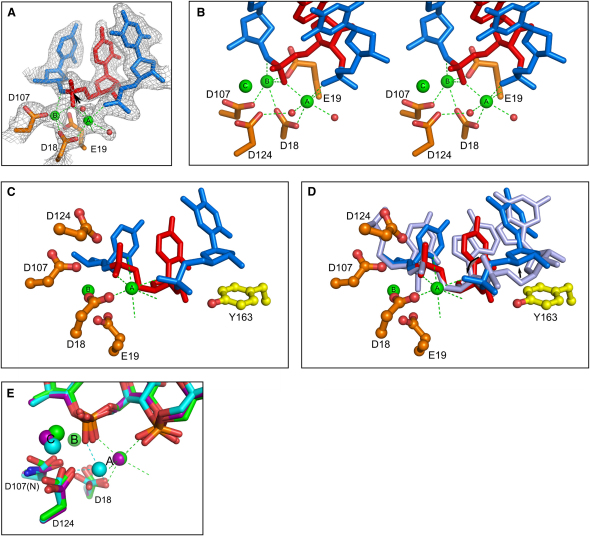
The Active Site (A) The active site with overlaid 2F_o_ − F_c_ simulated annealing omit electron density map contoured at 1 σ. The direction of the attack on the phosphorus atom by the putative nucleophile is shown with an arrow. RNA is shown in red and DNA in blue. Calcium ions and their coordination are shown in green. Water molecules are shown as red spheres. (B) Stereoview of the active site. (C) Close-up view of the coupling of the active site with the junction-sensing module. Active site residues are shown as orange ball and stick and tyrosine 163 as yellow ball and stick. Calcium ions are shown as green spheres. (D) Same view but with a fragment of ideal A-form RNA superimposed on the Tm-RNase H2 substrate to show the deformations induced at the (5′)RNA-DNA(3′) junction (indicated with arrows). (E) Superposition of structures solved in the presence of different metal ions. Metal ions are shown as spheres and the coordination of metal ion A as dashed lines. The structures are colored in green for wild-type protein with Ca^2+^, purple for D107N mutant protein structure solved in the presence of Mg^2+^, and cyan for D107N mutant protein with Mn^2+^.

The key element of the substrate interface is the recognition motif for the ribonucleotide at the (5′)RNA-DNA(3′) junction. It is recognized by a network of protein interactions formed with its 2′-OH group (Figure 4E). Backbone amide groups of R22 and glycines 21 and 23 located in a loop after strand 1 participate in these interactions. The GRG residues are conserved and form a bulge protruding into the minor groove. Another contact with the 2′-OH group is formed by the hydroxyl group of the Y163, located in the loop of the helix-loop-helix motif of the C-terminal domain (Figure 4E). The aromatic ring of the tyrosine stacks with the deoxyribose ring of the deoxyribonucleotide at the +2 position (numbering relative to the scissile phosphate) and seems to push it up (Figures 4E, 5C, and 5D), leading to deformation of the nucleic acid backbone at the (5′)RNA-DNA(3′) junction located between positions +1 and +2. The deformation is manifested by changes in γ and β angles of the phosphodiester backbone by around 100 degrees compared to the surrounding nucleotides or a regular double helix (Figures 5C and 5D). The stacking interaction between the ribose ring and the aromatic ring of tyrosine is efficient only if there is no 2′-OH group at position +2. Therefore, deoxyribonucleotide is preferred there. Together with 2′-OH detection at position +1 by GRG motif and Y163, this elegant mechanism assures the specific recognition and cleavage of an (5′)RNA-DNA(3′) junction.

### The Active Site

The active site of Tm-RNase H2 is composed of absolutely conserved carboxylates: D18 and E19 located at the end of strand 1, D107 from the C terminus of strand 4, and D124 from a loop before helix E (Figures 4D and 5A). Corresponding residues have been mutated in *A. fulgidus* and *T. kodakaraensis* RNases H2 (Chapados et al., 2001; Muroya et al., 2001). Mutations of equivalents of D18, E19, and D107 abolished the activity, while mutations of the equivalent of D124 led to a decrease in activity. In yeast RNase H2A, mutation of the residue corresponding to D18 (yeast D39) abolished the activity and mutations of equivalents of D107 and D124 (yeast D155 and D183) led to a loss of cleavage in RNA/DNA hybrid region, but (5′)RNA-DNA(3′) junction hydrolysis remained (Jeong et al., 2004).

The complex structure of wild-type protein solved in the presence of Ca^2+^ ions, which inhibit cleavage (structure WT-1R-Ca), contains three metal ions bound at the active site—two of them very similar in position to those observed in RNase H1 and other RISF enzymes. According to the RNase H1 nomenclature, these two ions are referred to as A and B and the third ion as C (Figure 5B). Ions A and C were also observed in structures of mutant Tm-RNase H2 D107N solved with Mg^2+^ (structure 1R-Mg), Mn^2+^ (structure 1R-Mn), and Ca^2+^ (data not shown), but metal ion B was missing in all these structures. Therefore, the lack of binding of ion B does not depend on its identity and is likely caused by D107N mutation. For RNase H1 substrate complex structures Mg^2+^ and Ca^2+^ ions occupy almost identical positions at the active site (Nowotny et al., 2005, 2007; Nowotny and Yang, 2006). Therefore, our WT-1R-Ca structure of RNase H2 with Ca^2+^ ions is likely a good model of the architecture of the catalytically competent active site with Mg^2+^ ions bound. We consequently assume that RNase H2 would utilize two-metal ion mechanism to perform nucleic acid hydrolysis.

The geometry of metal ion B coordination is quite irregular, as also observed in RNase H1 (Nowotny et al., 2005, 2007; Nowotny and Yang, 2006). It involves side chains of D18, E19, and D107 as well as two oxygens of the scissile phosphate—the 3′ leaving group and nonbridging pro-Sp (Figures 5A and 5B). The coordination of metal ion A is octahedral and quite regular, and the coordination distances are between 2.4 and 2.6 Å—longer than ideal distances for coordination of Mg^2+^ or Ca^2+^ (Harding, 2001). Metal ion A is coordinated by the side chain of D18, backbone carbonyl of E19, pro-Sp nonbridging oxygen of the scissile phosphate, and pro-Rp nonbridging oxygen of the phosphate group at position +2. Metal ion A also coordinates two water molecules. One of them is located 4.3 Å from the phosphorus atom of the scissile phosphate. This water molecule can be tentatively assigned as the attacking nucleophile, but it has to be brought much closer to the phosphorus atom to form the pentavalent transition state of the hydrolysis reaction (Nowotny and Yang, 2006). After the completion of the reaction, a product is formed with 5′ phosphate and 3′ OH groups.

The coordination of metal ion A by the phosphate group at position +2 is possible because of the deformation of the nucleic acid backbone introduced by the interaction with tyrosine 163 (Figures 5C and 5D). It appears to be the key element of a mechanism which assures substrate preference. Very likely, only the proper substrate containing the (5′)RNA-DNA(3′) junction can be deformed to coordinate metal ion A for optimal catalysis. Interestingly, in the structure solved after soaking of the 1R-Mg crystals with MnCl_2_, Mn^2+^ ion A is shifted and its coordination does not involve the phosphate group of the nucleotide at position +2. The nonbridging oxygen-metal ion distance is 3.3 Å for Mn^2+^ ion versus 2.4 Å for Mg^2+^ (Figure 5E). This may explain Mn^2+^-dependent hydrolysis of substrates other than (5′)RNA-DNA(3′) junctions by Tm-RNase H2 (Figure 2). Because these substrates contain a 2′-OH group in position +2, efficient stacking between Y163 and the ribose ring is not possible, and the deformed conformation of nucleic acid is likely different from the one observed in our structures. It might not allow the coordination of metal ion A by the phosphate at position +2. However, since binding of Mn^2+^ ion A does not involve a deformed +2 phosphate, Mn^2+^ can support cleavage of a suboptimal substrate. In general, the coordination requirements of Mn^2+^ are less stringent and many enzymes that depend on two-metal ion mechanism can act on atypical substrates in the presence of Mn^2+^ (Yang et al., 2006).

Metal ion C is located in the vicinity of residues D107 and D124 and has weaker electron density and higher B-factor, indicating that it is not very well organized. Metal ion C may play an auxiliary role in the reaction in a mechanism similar to the postulated three-metal ion hydrolysis of T5 FEN endonuclease (Syson et al., 2008), but its presence could also result from the growth of crystals at a relatively high divalent metal ion concentration.

**Figure 6:**
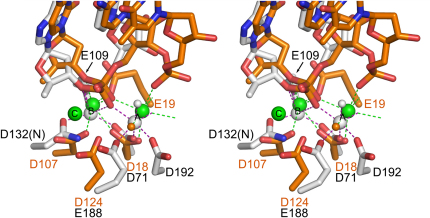
Comparison of the Active Sites of Type 1 and 2 RNases H Stereoview of the comparison of the active sites of *B. halodurans* RNase H1 (PDB 1ZBI) and *T. maritima* RNase H2. The Tm-RNase H complex is colored in orange (protein and nucleic acid) and green (Ca^2+^ ions) and Bh-RNase H1 in white. The attacking nucleophiles are shown as small spheres. Metal ion coordination is shown as dashed lines (green for Tm-RNase H2 and purple for Bh-RNase H1).

Metal ion coordination is quite well conserved among known nucleic acid complex structures of RISF proteins (Nowotny, 2009). Very similar positions of metal ions A and B have been observed in bacterial and human RNases H1, Tn5 transposase, and recently also in Argonaute (Wang et al., 2009). However, except for the two key carboxylates, D18 and D107, the active site of RNase H2 is different. The fourth carboxylate of the DEDD motif (D124 in Tm-RNase H2) has a very dissimilar position. Instead of being localized in the last helix of the RNase H fold (helix E), it is located in the loop preceding it. This carboxylate no longer coordinates metal ion A directly as in RNase H1, Tn5, and Argonaute, but through a water molecule (Figure 6). The coordination of metal ion A by this carboxylate is taken over by the distorted phosphate group at the position +2. D124 of Tm-RNase H2 is located in a similar position to E188 of *B. halodurans* RNase H1, which is a nonconserved auxiliary residue of the active site (Nowotny and Yang, 2006).

Metal ion B has a similar position and coordination between the two types of RNases H. In type 1 RNases H it involves the second carboxylate of the DEDD motif (E109 in *B. halodurans* RNase H1), which interacts with both the 2′-OH group of the ribonucleotide at position −1 and metal ion B. It was therefore proposed to serve as a substrate specificity check. In type 2 enzyme E19 is in similar position, even though it comes from a different part of the RNase H fold (Figure 6). It also coordinates Ca^2+^ ion B in wild-type Tm-RNase H2 structure. Based on modeling of a ribonucleotide at position −1, E19 can simultaneously coordinate metal ion B and form a hydrogen bond with a 2′-OH of nucleotide −1. Oligonucleotides with a single ribonucleotide do not have this 2′-OH group, making DNA_5_-RNA_1_-DNA_6_/DNA_12_ a slightly worse substrate for Tm-RNase H2 (Figure 2). During the cleavage of substrate with a single ribonucleotide, the conformation of E19 seems to be stabilized by R22.

In the vicinity of the active site, there is a loop between residues 41 and 51 that was not traced in the apo structure and in the complex has high B-factors and less well-defined electron density maps indicating mobility. It is inserted into the major groove of the substrate (Figure 3). Part of the loop is very well conserved, especially a DSK motif (residues 45–47 in Tm-RNase H2; Figures 3 and 4D). K47 interacts with the pro-Rp nonbridging oxygen of the scissile phosphate, and D45 and S46 are around 3.1–3.3 Å away from the putative attacking nucleophile. Therefore, DSK motif might participate in the active site formation.

### Comparison with Type 3 RNase H

The third, less common class of RNases H are type 3 enzymes or RNases H3. They are present in some bacteria and archaea (Kochiwa et al., 2007). Based on amino acid sequence and structure, RNases H3 are quite similar to type 2 enzymes—when structures of Tm-RNase H2 and *B. stearothermophilus* RNase H3 (Chon et al., 2006) are superimposed using the conserved elements of the catalytic domain, the rmsd is 1.46 Å over 100 Cα atoms. Several nucleic acid-interacting residues are conserved including the glycines of the GRG motif. There is one very important difference, however—the tyrosine at the heart of the (5′)RNA-DNA(3′) junction recognition module is not present in type 3 enzymes. This explains, at least in part, why RNases H3 fail to cleave (5′)RNA-DNA(3′) junction substrates (Ohtani et al., 1999, 2008) and thus resemble type 1 enzymes in biochemical properties.

### Enzymatic Activities of Mutant Proteins of Tm-RNase H2

**Figure 7:**
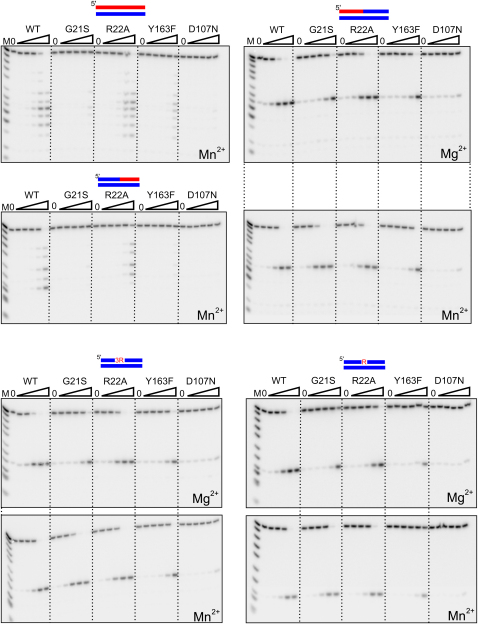
Biochemical Characterization of Mutant Tm-RNase H2 Proteins The 5′ end ^32^P-labeled substrates (1 μM) indicated on top of the gels (RNA in red, DNA in blue) were hydrolyzed with increasing concentrations of Tm-RNase H2 (wild-type and the derivatives). Lanes indicated by 0 contained no enzyme and those indicated by triangle increasing amounts of the protein (0.016, 0.16, 1.6, 16, and 160 nM). Reactions (20 μl) were incubated at 37°C for 30 min in the presence of metal ions indicated in the bottom of each gel. The sizes of products were measured based on molecular size markers indicated as M (products of digestion of ^32^P-labeled strands without complementary DNA by phosphodiesterase I). Products of the hydrolysis were analyzed by 20% TBE-urea polyacrylamide gels.

To further understand the mechanism of RNase H2, we introduced G21S, Y163F, and R22A mutations to Tm-RNase H2 and studied the activity of mutant proteins on different substrates (Figure 7). D107N active site mutant that shows no enzymatic activity was used as a negative control. Y163 is a key residue involved in 2′-OH binding, and indeed, its mutation to phenylalanine seriously reduced activity against all tested substrates. Therefore, Y163 is important for binding of both (5′)RNA-DNA(3′) junctions and RNA/DNA substrates.

G21S mutant corresponds to the mutation observed in human RNase H2A protein in AGS patients and is located in the conserved GRG 2′-OH-sensing motif. In the human protein, glycine to serine mutation leads to a significant reduction in enzymatic activity on RNA/DNA hybrids and substrates with a single ribonucleotide (Chon et al., 2009; Crow et al., 2006; Perrino et al., 2009). Tm-RNase H2 G21S likewise has much lower activity on RNA/DNA and DNA-RNA/DNA in the presence of Mn^2+^. In the presence of Mg^2+^ it also showed reduced activity on substrates with (5′)RNA-DNA(3′) junction (Figure 7). This observation differs from that reported for human RNase H2A G37S in which junction cleavage in RNA-DNA/DNA duplexes remains intact while RNA/DNA cleavage is lost (Chon et al., 2009; Crow et al., 2006; Perrino et al., 2009). Similar results to ours have been reported for a corresponding mutant enzyme in yeast and *T. kodakareansis* RNase H2 (Rohman et al., 2008). Glycine to serine mutation must introduce steric clashes with the neighboring residues. To alleviate conflicts, the backbone of the protein has to change its conformation. Since this protein region forms very tight contacts with the substrate, its conformation is also likely to be altered. However, in the presence of Mn^2+^, Tm-RNase H2 G21S cleaved substrates with (5′)RNA-DNA(3′) junctions as efficiently as the wild-type protein. In general, cleavages in RNA/DNA regions of the substrate were much more affected than the cleavage of the (5′)RNA-DNA(3′) junction. The deformation of (5′)RNA-DNA(3′) junction and (5′)RNA-RNA(3′) substrates at the active site are likely to be different. For (5′)RNA-DNA(3′) junctions, G21S might introduce only a slight displacement of deformed phosphate, so that it is no longer able to coordinate metal ion A. Mg-dependent activity is reduced, but since Mn^2+^ does not require this additional coordination, it supports wild-type levels of activity. For (5′)RNA-RNA(3′) cleavage G21S might introduce a more pronounced displacement, which would also involve the scissile phosphate, such that there is no cleavage even in the presence of Mn^2+^.

R22A mutation, which should affect the interactions with the minor groove of the substrate, does not alter Tm-RNase H2 activity on most substrates. It leads to a decrease in cleavage of DNA_5_-RNA_7_/DNA_12_ hybrid at the (5′)DNA-RNA(3′) junction. It also caused a small decrease in the cleavage of the substrate with a single ribonucleotide (DNA_5_-RNA_1_-DNA_6_/DNA_12_). This substrate lacks a 2′-OH group at position −1, which may interact with and stabilize the conformation of the active site residue E19 involved in coordination of metal ion B. In wild-type protein the stabilization of E19 is taken over by R22, and since its side chain is missing in R22A mutant, its activity is decreased.

### Implications for AGS Mutant Enzymes

While this work was being prepared, a structure of a complex of mouse RNase H2 containing subunits A, B, and C was published (Shaban et al., 2010). Tm-RNase H2 and mouse RNase H2A are almost identical in structure. When they are superimposed using the most conserved secondary structure elements (central β sheet and helices B and E), the rmsd over 68 C-α atoms is 0.98 Å. Human and mouse enzymes are 80% identical in sequence of the catalytic subunit, and their structures must be very similar. There are significant differences between *T. maritima* and eukaryotic RNases H2; most importantly, human and yeast enzymes have much higher Mg-dependent activity on RNA/DNA hybrids. However, given the structural similarity and the conservation of residues essential for substrate binding, the cleavage mechanism is likely to be similar in bacterial and eukaryotic enzymes (Figure 4D). Therefore, some predictions can be made about the mutations observed in AGS in the catalytic subunit of human RNase H2 (Crow et al., 2006; Rice et al., 2007). For the human enzyme, the G37S AGS-causing mutation affects the cleavage of RNA/DNA and DNA-RNA_1_-DNA/DNA substrates, but not the RNA-DNA/DNA junction of Okazaki-fragment model substrate (Chon et al., 2009; Crow et al., 2006; Perrino et al., 2009; Shaban et al., 2010). It suggests that the inability to cleave regular RNA/DNA hybrids and/or single ribonucleotides embedded in the DNA is the defect in these AGS patients. Other mutations found in AGS patients localize to residues R186 and R235, which correspond to R141 and R182 in Tm-RNase H2, respectively (Rice et al., 2007). Both are structural residues which stabilize the GRG motif for 2′-OH binding. Another reported mutation is in residue T240. Its structural equivalent in Tm-RNase H2 is P187, which forms van der Waals interactions with DNA backbone. Therefore, its mutation would affect noncleaved strand binding.

### Conclusions

We described structures of Tm-RNase H2 in complex with substrates containing a (5′)RNA-DNA(3′) junction specifically cleaved by this enzyme. They reveal a mechanism of the junction sensing which involves specific contact with the 2′-OH group and is coupled to the coordination of the catalytic metal ion. This simple and elegant substrate-assisted mechanism allows for specificity of cleavage. The recognition of (5′)RNA-DNA(3′) junction is thought to be important for the removal of RNA embedded in the DNA and hence would be a key DNA repair mechanism. Our structures also provide a more complete picture of the two types of RNases H and help explain the differences in their biochemical properties.

## Experimental Procedures

### Crystallization

The detailed description of Tm-RNase H2 preparation can be found in the Supplemental Information. Briefly, the protein was expressed in *E. coli* and purified by heating of the extract, hydrophobic interaction chromatography, and gel filtration.

HPLC-purified oligonucleotides were purchased from Metabion (Martinsried, Germany) and IDT (Coralville, IL, USA). For crystallization, 1R oligo was produced by annealing the cleaved strand (5′-GACACcTGATTC; single ribonucleotide, small caps). with the complementary DNA strand (5′-GAATCAGGTGTC).

Prior to crystallization, Tm-RNase H2 D107N ΔC (15 residues removed from the C terminus) was mixed with the oligos at 1.2:1 substrate:protein molar ratio. The final protein concentration was 2 mg/ml. The complexes were mixed with the reservoir solution at equal volume and crystallized by the sitting drop vapor diffusion method at 18°C. The 1R-Mg^2+^ complex crystals were obtained with 0.3 M MgCl_2_, 23% PEG 3350, and 0.1 M HEPES (pH 7.5). For data collection the crystals were transferred to cryoprotecting solution, which contained the well solution but with 35% PEG 3350. The 1R-Mn^2+^ crystals were obtained by soaking 1R-Mg^2+^ crystals with MnCl_2_. The crystals were transferred stepwise to soaking solutions with increasing MnCl_2_, NaCl, and PEG 3350 concentrations and decreasing MgCl_2_ concentration. The final solution contained 30 mM MnCl_2_, 0.2 M NaCl, 35% PEG 3350, and 0.1 M HEPES (pH 7.5). The wild-type crystals were obtained with 0.3 M CaCl_2_, 0.1 M Tris-HCl (pH 8.5), 20% PEG 3350. The cryoprotecting solution contained increased concentration of PEG 3350 (35%). All crystals were flash frozen in liquid nitrogen.

### Data Collection and Structure Determination

The diffraction data of the 1R-Mg crystals were collected on Berliner Elektronenspeicherring-Gesellschaft für Synchrotronstrahlung (BESSY) synchrotron at beamline MX-14.2 on a Mar225 CCD detector at 100 K (Table 1). The 1R-Mn data set was collected at BW6 beamline at Deutsches Elektronen Synchrotron (DESY) on a Mar165 detector. The WT-1R-Ca data set was collected at 14-4 beamline at European Synchrotron Radiation Facility (ESRF). The data sets were processed and scaled using HKL2000 (Otwinowski and Minor, 1997) (Table 1). The structures belong to C2 spacegroup and contain one complex in the asymmetric unit. The first complex structure was solved using molecular replacement method using the apo protein structure (PDB 2ETJ) and Phaser program (McCoy et al., 2007). The model for nucleic acid was built manually in Coot (Emsley and Cowtan, 2004) to produce the complete model of the complex. The subsequent structures were solved using molecular replacement with the complex model. The resulting model was refined using phenix.refine (Afonine et al., 2005) interspersed with manual building in Coot. All the protein and nucleic acid residues can be traced in the three structures.

The nucleic acid conformation was analyzed using program CURVES+ (Lavery et al., 2009). Structure analyses including superpositions were done in Pymol (http://www.pymol.org/). Surface potentials were calculated with APBS (Baker et al., 2001). Figures were prepared using Pymol.

### RNase H Cleavage Assays

RNase H activity was determined using a uniformly γ^32^P-ATP-labeled poly(rA)/poly(dT) substrate by measuring the amount of radioactivity of the acid-soluble digestion product in various solution conditions as described previously (Gaidamakov et al., 2005). For assays with Mg^2+^, the enzyme concentration was 40 and 4 nM, and for assays in the presence of Mn^2+^, 0.4 and 0.04 nM. For short substrate experiments, the 5′-^32^P-labeled 12-mer RNA/DNA (the sequences of the oligonucleotides are given in Figure 2) were digested with native and mutant proteins in 15 mM Tris-HCl (pH 7.9), 50 mM NaCl, 1 mM DTT, 100 μg/ml BSA, 5% glycerol, and 1 mM MgCl_2_ (or 1 mM MnCl_2_). Products of hydrolysis were analyzed by 20% TBE-urea polyacrylamide gels. The reaction products were visualized by phosphorimaging.

## Figures and Tables

**Table 1:** Data Collection and Refinement Statistics

Structure	Tm-RNase H2 ΔC	Tm-RNase H2 ΔC	Tm-RNase H2 ΔC
	D107N	CaCl_2_	D107N
	MgCl_2_	(WT-1R-Ca)	MnCl_2_
	(1R-Mg)		(1R-Mn)
**Data Collection**			

Space group	C2	C2	C2
Cell dimensions			
a, b, c (Å)	105.06, 48.57, 78.39	106.12, 47.35, 79.01	104.66, 48.57, 77.91
α, β, γ	90, 131.80, 90	90, 132.81, 90	90, 131.97, 90
Resolution (Å)	30–2.0 (2.03–2.00)^a^	50–2.1 (2.14–2.10)^a^	50–2.8 (2.85–2.80)^a^
R_merge_ (%)	6.4 (42.4)	8.6 (48.4)	8.4 (33.0)
I/σI	21.6 (1.9)	20.5 (2.8)	21.0 (2.5)
Completeness (%)	94.9 (68.4)	99.2 (98.8)	93.3 (57.4)
Redundancy	3.4 (2.1)	3.6 (3.4)	6.2 (2.6)

**Refinement**			

Resolution (Å)	2.0	2.1	2.8
Number of reflections	17,874	15,873	6,616
R_free_ set	943 (5.28%)	816 (5.14%)	646 (9.76%)
R_work_/R_free_ (%)	19.1/24.4	17.1/24.5	18.2/25.3
Number of atoms	2,374	2,471	2,276
Protein	1,739	1,735	1,738
Nucleic acids	487	487	486
Water	146	244	49
Metal ions	2	5	3
B-factors	37.6	38.7	32.2
Protein	36.8	35.9	32.1
Nucleic acids	40.0	44.4	32.8
Water	39.0	47.1	28.8
Metal ions	44.4	44.5	49.2
Rmsds			
Bond lengths (Å)	0.007	0.008	0.007
Bond angles (°)	1.163	1.254	1.168

a Values in parentheses are for highest-resolution shell.

## Accession Numbers

The 1R-Mg, 1R-Mn, and WT-1R-Ca structures have been deposited in the Protein Data Bank under accession codes 3O3F, 3O3H, and 3O3G, respectively.

## Supplemental Information

Document S1. Supplemental Experimental Procedures
