# Supplementary material for: Crystal Structures of RNase H2 in Complex with Nucleic Acid Reveal the Mechanism of RNA-DNA Junction Recognition and Cleavage
Source: Mol Cell. 2010 Nov 24;40(4):658–70. doi: 10.1016/j.molcel.2010.11.001 (PMC3025331; doi:10.1016/j.molcel.2010.11.001)
Supplement: Document S1. Supplemental Experimental Procedures [file mmc1.pdf]

Molecular Cell, Volume *40*

Supplemental Information

Crystal Structures of RNase H2 in Complex with Nucleic Acid Reveal the Mechanism of RNA-DNA Junction Recognition and Cleavage

Monika P. Rychlik, Hyongi Chon, Susana M. Cerritelli, Paulina Klimek, Robert J. Crouch, and Marcin Nowotny

Supplemental Experimental Procedures

Protein preparation

The Tm-RNase H2 expression plasmids were prepared based on pET21 expression vector (Novagen). The sequence coding for the protein was inserted into NdeI and EcoRI restriction sites, so that the expressed protein did not contain any tags. The mutagenesis of the construct was carried out using the QuikChange kit (Stratagene).

All four variants of Tm-RNase H2 protein (wild-type and D107N, both in full length and Δ15C form) were expressed in *E. coli* strain BL21 using the induction with 0.4 mM IPTG. Bacterial cell cells were resuspended in 40 mM NaH_2PO_4 (pH 7.0), 75 mM NaCl, 5% glycerol, 1 mM DTT, 0.5 mM EDTA with the addition of a mix of protease inhibitors. Next, lysozyme and Benzonase (Novagen) were added to 1 μg/ml and 250 U/ml final concentration, respectively. After incubation on ice and sonication, the cleared lysate was heated at 70°C for 10 minutes to denature and precipitate the host proteins. NH_4SO_4 was added to the supernatant to a final concentration of 2 M and it was applied to a Resource Phenyl column (GE Healthcare) equilibrated with 2 M NH_4SO_4, 40 mM NaH_2PO_4, 5% glycerol, 1 mM DTT, and 0.5 mM EDTA. The protein was eluted with a linear gradient of NH_4SO_4 from 2 M to 0 M. Fractions containing the protein were concentrated and applied to a gel filtration column (Superdex 75, GE Healthcare) to remove the aggregate containing non-specifically bound nucleic acid. The protein was concentrated to 2.4 – 3 mg/ml and stored in 20 mM HEPES (pH 7.0), 75 mM NaCl, 5% glycerol, 0.1 mM DTT, 0.5 mM EDTA.
